# Occupational exposure to carbon black nanoparticles increases inflammatory vascular disease risk: an implication of an ex vivo biosensor assay

**DOI:** 10.1186/s12989-020-00378-8

**Published:** 2020-09-29

**Authors:** Jinglong Tang, Wenting Cheng, Jinling Gao, Yanting Li, Ruyong Yao, Nathaniel Rothman, Qing Lan, Matthew J. Campen, Yuxin Zheng, Shuguang Leng

**Affiliations:** 1 Department of Occupational and Environmental Health, School of Public Health, Qingdao University, Qingdao, 266021 China; 2 Department of Central Laboratory, Affiliated Hospital of Medical College of Qingdao University, Qingdao University, Qingdao, 266021 China; 3 Division of Cancer Epidemiology and Genetics, National Cancer Institute, National Institutes of Health, Rockville, MD USA; 4 Department of Pharmaceutical Sciences, College of Pharmacy, University of New Mexico, Albuquerque, 87131 USA; 5 Department of Internal Medicine, School of Medicine, University of New Mexico, Albuquerque, NM 87131 USA; 6 Cancer Control and Population Sciences, University of New Mexico Comprehensive Cancer Center, Albuquerque, NM 87131 USA

**Keywords:** Carbon black nanoparticles, Biosensor, Endothelial cell activation, Mediation effect

## Abstract

**Background:**

Among manufactured or engineered nanoparticles, carbon black (CB) has largest production worldwide and is also an occupational respiratory hazard commonly seen in rubber industry. Few studies have assessed the risk for cardiovascular disease in carbon black exposed populations. An endothelial biosensor assay was used to quantify the capacity of sera from 82 carbon black packers (CBP) and 106 non-CBPs to induce endothelial cell activation ex vivo. The mediation effect of circulatory proinflammatory factors on the association between carbon black exposure and endothelial cell activation was assessed and further validated using in vitro intervention experiments.

**Results:**

The average elemental carbon level inside carbon black bagging facilities was 657.0 μg/m^3^, which was 164-fold higher than that seen in reference areas (4.0 μg/m^3^). A global index was extracted from mRNA expression of seven candidate biosensor genes using principal component analysis and used to quantify the magnitude of endothelial cell activation. This global index was found to be significantly altered in CBPs compared to non-CBPs (*P* < 0.0001), however this difference did not vary by smoking status (*P* = 0.74). Individual gene analyses identified that de novo expression of key adhesion molecules (e.g., ICAM and VCAM) and chemotactic factors (e.g., CCL2, CCL5, and CXCL8) responsible for the recruitment of leukocytes was dramatically induced in CBPs with CXCL8 showing the highest fold of induction (relative quantification = 9.1, *P* < 0.0001). The combination of mediation analyses and in vitro functional validation confirmed TNF-α, IL-1β, and IL-6 as important circulatory factors mediating the effects of carbon black exposure on endothelial cell activation responses.

**Conclusions:**

Inflammatory mediators in sera from CBPs may bridge carbon black exposure and endothelial cell activation response assessed ex vivo. CBPs may have elevated risk for cardiovascular diseases when comorbidity exists. Our study may serve as a benchmark for understanding health effects of engineered carbon based nanoparticles with environmental and occupational health relevance.

## Introduction

Among manufactured or engineered carbon containing nanoparticles, carbon black (Chemical Abstracts Service Registry No. 1333-86-4) has the highest production worldwide and consists of pure elemental carbon [1–3]. Carbon black has been recognized as an occupational respiratory hazard commonly seen in industries that manufacture it or apply it in the formulation of rubber, printing inks, and paints. Extensive epidemiological and animal studies have established a causal link between exposure to air pollution of inhalable particulate matters and morbidity and mortality of cardiovascular diseases [4–6]. Several studies have also found that carbon black exposure may impair vasomotor function and accelerate plaque area development in the aorta of genetically manipulated mice susceptible to the development of atherosclerosis [7, 8]. However, although carbon black has been used as a carbonaceous core analog of airborne particulate matters in many in vitro and in vivo animal studies [9, 10], evidence supporting its cardiovascular toxicity in humans is still inconclusive [11].

As one category of poorly soluble nanoparticles, the main health concerns in humans associated with carbon black exposure are lung effects resulting from inhalation exposure [12–15]. However, we found dramatically elevated multiple pro-inflammatory cytokines and chemokines in serum of carbon black packers (CBP) and in male BALB/c mice exposed to carbon black aerosol [14]. Moreover, our study identified elevated genomic instability in peripheral lymphocytes in CBPs that could be mediated mostly by circulatory TNF-α [16], suggesting a systemic inflammation mediated mechanism for extra-pulmonary effects of carbon black inhalation exposure. Compelling evidence supports inflammation processes that pivotally participate in all stages of atherosclerosis as a bridge linking classic risk factors to altered cellular behavior within the arterial wall. Many circulatory inflammatory markers have been identified in clinical studies to be associated with propensity for developing ischemic events and prognosis after acute coronary syndromes [17, 18]. However, although elevated inflammation was observed in the circulation in our cohort of CBPs, the young age of this population (average age 45.7 years) made it impossible to observe any incidence of cardiovascular diseases.

Vascular disease is an inflammatory condition wherein activated endothelial cells mediate the recruitment of immune cells into damaged areas of the vessel wall, promoting oxidative injury, pathological lesion growth, and histological complexity [19]. Endothelial cell activation, often induced by cytokines such as CRP and TNF-α via the nuclear factor-kappa beta pathway, leads to de novo expression or presentation of key adhesion molecules and chemotactic factors in cell surfaces for the recruitment of leukocytes [20]. Dr. Campen’s group has developed a novel endothelial biosensor assay in which the capacity of a serum sample to induce endothelial cell activation is quantified by assessing mRNA expression of key adhesion molecules and chemotactic factors in primary human coronary artery endothelial cells (hCAEC) treated ex vivo with diluted serum samples from study subjects. This method has been validated by showing a greater potential for inducing de novo expression of endothelial cell adhesion molecules and chemokines in hCAECs treated with serum samples from patients with coronary artery disease or obstructive sleep apnea [21, 22]. Moreover, in a controlled diesel engine exhaust exposure study, serum from human subjects was found to exhibit greater inflammatory bioactivity on cultured endothelial cells compared to serum obtained following sham exposures; while nitrogen dioxide exposure recapitulated the early inflammatory bioactivity, the inflammatory effect of diesel engine exhaust was sustained for at least 24 h after exposure [23].

In this study, we used the endothelial biosensor assay to assess holistic inflammatory potential in serum samples from 82 CBPs and 106 non-CBPs. We hypothesized that exposure to carbon black aerosol could increase endothelial cell activation ex vivo through elevating circulatory levels of proinflammatory factors. The findings would provide strong evidence supporting the effect of carbon black exposure on the risk of developing cardiovascular diseases in carbon black exposed populations and may serve as a benchmark for understanding health effects of engineered carbon based nanoparticles with environmental and occupational health relevance.

## Results

### Physicochemical characteristics of carbon black particles

Physicochemical characteristics of carbon black particles have been assessed extensively in reference [14]. A thermal decomposition of acetylene technique was used to manufacture carbon black in the studied facility. Accordingly, carbon black product from this facility has a very high carbon purity (> 99.8%). Under a transmission electron microscope, the primary carbon black unit consists of globular shaped particles with diameters ranging from 30 to 50 nm. A scanning electron microscope identified formation of aggregates and agglomerates with hundreds to thousands of nanometers in at least one dimension and forming an aciniform morphology [14]. Carbon black has a surface area of 74.85 m^2^/g measured using the Brunauer-Emmett-Teller method. Carbon black suspension in water has a zeta potential of − 15.37 mV.

### Characterization of study subjects and carbon black aerosol in working environment

All study subjects were male with Han Chinese ethnicity. The distribution of age, overweight or obesity, and smoking history was similar in CBP and non-CBP control groups (all Ps > 0.065, Table 1). Because acetylene carbon black is almost pure elemental carbon [14], the summed level of urinary hydroxyl polycyclic aromatic hydrocarbons was not different between CBPs and non-CBPs. The average level of particulate matter with diameters of 2.5 μm or less (PM2.5) in carbon black bagging areas was 800.0 μg/m^3^ with elemental carbon level at 657.0 μg/m^3^ (Table 1) [14, 24], well within the recommended long term exposure limit of carbon black (3.5–4 mg/m^3^) in North America, European Union, and China [25]. Size distribution analysis of carbon black aerosol inside the bagging areas showed that up to 99.6% of the carbon black particles had aerodynamic diameter less than 2.5 μm with 96.7% of the particulates less than 1.0 μm [14].

**Table 1:** Demographics of 106 non-CBPs and 82 CBPs

Variable	Non-CBP	CBP	*P* value
n	106	82	
Age (y, M ± SD)	44.2 ± 5.8	45.7 ± 5.3	0.065^a^
Sex (Male, %)	100	100	
Race (Han Chinese, %)	100	100	
Current smokers (n, %)	69, 65.1	57, 69.5	0.52^b^
Packyears (Mdn, Q1-Q3)^d^	13.5 (6.0–25)	10 (8–20)	0.34^c^
Overweight or obesity (n, %)	52, 49.1	33, 40.2	0.23^b^
Urinary OH-PAHs (μg/g cr, Mdn, Q1-Q3)^e^	6.34 (2.97–10.92)	4.95 (3.22–7.76)	0.35^c^
PM_2.5_ (μg/m^3^, M ± SD, n)	71.0 ± 11.4, 5	800.0 ± 574.5, 16	
EC (μg/m^3^, M ± SD, n)	4.0 ± 0.2, 5	657.0 ± 73.7, 16	
Total RNA (μg, Mdn, Q1-Q3)	0.82 (0.47–1.62)	0.75 (0.44–1.69)	0.65^c^

*CBP* Carbon black packer, *Q* Quartile, *M* Mean, *Mdn* Median, *PM* Particulate matter, *EC* Elemental carbon, *SD* Standard deviation

^a^Student t test

^b^Chi square test

^c^ Wilcoxon Rank sum test

^d^Values in current smokers

^e^Urinary OH-PAHs were the sum of 1-hydroxynaphthalene, 2-hydroxynaphthalene, 2-hydroxyfluorene, 2-hydroxyphenanthrene, 9-hydroxyphenanthrene, and 1-hydroxypyrene in urine

### Effect of carbon black exposure on hCAEC activation

All biosensor genes except CCL5 and SELP had mRNA abundance substantially higher than the endogenous control gene (TBP, Table 2). Principal component analysis based on delta Ct of mRNA expression of 7 genes identified a major principal component (i.e., biosensor PC1) that explained 41% of total variance and had positive loading from delta Ct of all 7 genes with major loadings (score > 0.3) from CCL2, CCL5, CXCL8, ICAM, and VCAM (Supplemental Table 1). Thus, we defined biosensor PC1 as a global score for hCAEC activation. Biosensor PC1 was significantly reduced in cultures treated with sera from CBPs versus non-CBPs (i.e., estimate = − 0.92), indicating increased mRNA expression (i.e., relative quantification = 1.89) of the 5 inflammatory genes mentioned above (Table 2). Findings from individual gene association analyses substantiated results from the above analyses with CXCL8 having the largest fold of increase in expression (relative quantification = 9.06, *P* < 0.0001, Table 2). An interaction term between smoking status and carbon black exposure was included in the generalized linear model to assess whether the association between carbon black exposure and endothelial cell activation (i.e., biosensor PC1) varied by smoking status. The *P* value for the interaction term was 0.72 (not shown), suggesting smoking status did not affect the association between carbon black exposure and endothelial cell activation. Carbon black exposure history was not associated with biosensor PC1 (*P* = 0.35, not shown).

**Table 2:** mRNA expression of seven genes in primary hCAECs treated with sera from 106 non-CBPs and 82 CBPs^a^

Gene	Non-CBP	CBP	Estimate (95%CI)	RQ (95%CI)^b^	P
Biosensor PC1	0.62 (− 0.27–1.60)	− 0.26 (− 0.92–0.17)	− 0.92 (− 1.31 – − 0.53)	1.89 (1.44–2.48)	< 0.0001
CCL2	−7.10 (−8.05 – − 6.35)	−7.67 (− 8.47 – − 7.15)	− 0.57 (− 1.00 – − 0.13)	1.48 (1.09–2.00)	0.011
CCL5	− 0.76 (− 1.27 – − 0.28)	−1.29 (− 1.94 – − 0.74)	− 0.48 (− 0.77 – − 0.19)	1.39 (1.14–1.71)	0.0012
CXCL8	−7.05 (− 9.33 – −5.10)	− 10.29 (− 12.11 – − 8.26)	−3.18 (− 4.01 – − 2.35)	9.06 (5.10–16.11)	< 0.0001
CXCL12	− 2.63 (− 3.30 – − 2.21)	−2.33 (− 2.65 – − 1.99)	0.62 (0.23–1.01)	0.65 (0.50–0.85)	0.0018
ICAM	−4.56 (− 5.96 – − 3.83)	−4.96 (− 5.77 – − 4.42)	− 0.39 (− 0.72 – − 0.06)	1.31 (1.04–1.65)	0.021
SELP	0.07 (− 0.32–0.46)	− 0.02 (− 0.32–0.37)	− 0.15 (− 0.47–0.18)	1.11 (0.88–1.39)	0.37
VCAM	−1.71 (− 2.66 – − 0.94)	−2.59 (− 3.15 – − 1.84)	− 0.69 (− 1.14 – − 0.24)	1.61 (1.18–2.20)	0.0027

*CBP* Carbon black packer, *RQ* Relative quantification, *CI* Confidence interval, *hCAEC* Human coronary artery endothelial cell, *GLM* Generalized linear model

^a^Biosensor PC1was the major principal component extracted from delta Cts of the seven genes. Delta Cts of target genes relative to endogenous control gene (TBP) were expressed as median and interquartile range. Estimates were calculated based on biosensor PC1 or delta Ct of individual genes using GLM with adjustment for age, overweight and obesity, current smoking status, packyears, and passage of cells

^b^Relative quantification was calculated as RQ = 2^-(estimate)^

### Correlation between mRNA expression and protein secreted in culture medium for CXCL8

Every 2-fold increase in mRNA expression of CXCL8 was associated with 41.4 pg/ml (95%CI = 16.3–66.6 pg/ml, *P* = 0.0018, *n* = 52) increase in net CXCL8 protein levels in supernatants using a generalized linear model with adjustment for age, overweight or obesity, current smoking status, packyears, passage of cells, and batch of assay. Net increase of CXCL8 protein level was also significantly (*P* < 0.0001) higher in cultures spiked with serum from CBPs (least square mean = 820.8 with 95%CI as 723.5–918.1, *n* = 19) versus non-CBPs (least square mean = 145.2 with 95%CI as 79.8–210.6, *n* = 33).

### Circulatory inflammation mediating the effect of carbon black exposure on hCAEC activation

**Fig. 1:**
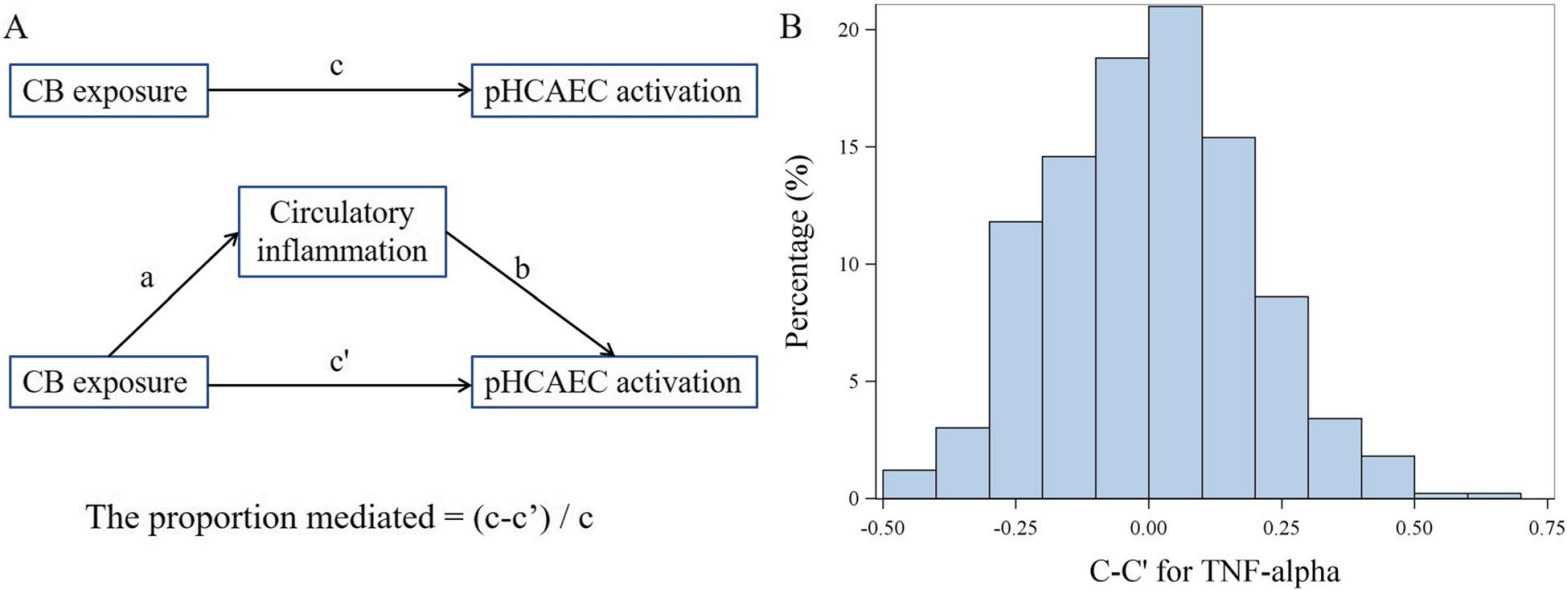
Circulatory inflammation mediating carbon black exposure induced ex vivo hACEC activation. In mediation analysis (**a**), the c coefficient denotes the direct effect of carbon black exposure on hACEC activation, without controlling for circulatory inflammation (mediator). The c’ coefficient denotes the direct effect of carbon black exposure on hACEC activation, controlling for circulatory inflammation (mediator). The proportion mediated is equal to delta c (i.e., c-c’) divided by c. We used a permutation-based method to assess if the proportion mediated was statistically significant (**b**). The relationship between biosensor PC1 and the vector of independent variables was permuted for 500 times. Each permutated dataset allowed for the association analysis of biosensor PC1 with carbon black exposure and other covariates without and with including TNF-α to calculate the c and c’. Permutation was conducted for 500 times to generate the distribution of c-c’ under null hypothesis of no mediation. Value of c-c’ calculated using observed data (− 0.768) was compared to the distribution generated by permutation and Pperm was calculated as the number of permuted databases generating a c-c’ that is smaller than observed value (*n* = 0 for TNF-α) divided by 500. CB = carbon black; pHCAEC = primary human coronary artery endothelial cell

Our previous study identified that carbon black exposure increased levels of multiple circulatory pro-inflammatory factors [14]. We quantified such differences and found that TNF-α, IL-1β, and IL-6 exhibited the greatest increases (7.0- to 10.6-fold) followed by MIP-1β (3.3-fold) and CRP (2.7-fold, Supplemental Table 2). Because several of these factors are known inflammatory mediators and established risk factors for cardiovascular diseases [23], mediation analyses were conducted to assess whether the increased potency of inducing mRNA expression of biosensor genes due to carbon black exposure could be mediated by these 5 factors in serum (Fig. 1a). Analyses provided evidence to support all 5 factors as mediators for the association between carbon black exposure and biosensor PC1, with TNF-α having a complete mediation effect (Table 3, Fig. 1b). Mediation analyses were further conducted with individual gene expression (delta Ct) as the outcome (Table 4). Among all mediation effects identified, serum TNF-α and IL-6 completely negated the association between carbon black exposure and increased gene expression of ICAM, VCAM, CCL2, and CCL5 and partially mediated the association between carbon black exposure and increased CXCL8 expression. Evidence of mediation effects was also identified for circulatory IL-1β, MIP-1β, and CRP, but to a much lesser degree.

**Table 3:** Pattern of triangles between carbon black exposure, pro-inflammation level, and biosensor responses in 106 non-CBPs and 82 CBPs

Variable	IQR in non-CBPs	Factor – biosensor association^a^			Carbon black – biosensor association^a^			Mediation effect^b^	
		Estimate	SE	P	Estimate	SE	P	PM	P perm
Carbon black exposure					−0.920 (c)^c^	0.198	< 0.0001		
IL-6 (pg/ml)	52.1	−0.134 (b)	0.040	0.0010	−0.480 (c’)	0.233	0.041	0.478	0.014
TNF-α (pg/ml)	94.3	−0.422 (b)	0.078	< 0.0001	−0.152 (c’)	0.232	0.51	0.835	< 0.002
MIP-1β (ng/ml)	1.05	−0.130 (b)	0.044	0.0033	−0.600 (c’)	0.222	0.0076	0.348	0.02
IL-1β (pg/ml)	6.2	−0.090 (b)	0.030	0.0027	−0.798 (c’)	0.198	< 0.0001	0.133	0.03
CRP (mg/L)	0.75	−0.055 (b)	0.028	0.0487	−0.812 (c’)	0.204	0.0001	0.117	0.082

*CBP* Carbon black packer, *IQR* Inter-quartile range, *SE* Standard error, *GLM* Generalized linear model

^a^GLM was used to assess the association (c’) between carbon black exposure and biosensor PC1 with age, overweight and obesity, current smoking status, packyears, passage of cells, and cytokines or chemokines (b) included as covariate for adjustment. Non-transformed data of cytokine and chemokine levels were used in GLM. c’ and b should be referred to Fig. 1

^b^The proportion mediated effect size that quantifies the proportion of a total effect mediated was calculated using the following equation: (c-c’) / c. The database was permuted for 500 times to generate a null distribution of c-c’ (Fig. 1b). P_perm_ was calculated as the number of permuted databases generating a c-c’ that is less than observed value divided by 500. c should be referred to Fig. 1

^c^GLM was used to assess the association (c) between carbon black exposure and biosensor PC1 in 106 non-CBPs and 82 CBPs with adjustment for age, overweight and obesity, current smoking status, and packyears, and passage of cells. No cytokines or chemokines were included in this model. c should be referred to Fig. 1

**Table 4:** Mediation effect of TNF-α, IL-1β, and IL-6 on individual biosensor genes in 106 non-CBPs and 82 CBPs^a^

Biosensor	Variable	IQR in non-CBPs	Factor – biosensor association^a^			Carbon black – biosensor association^a^			Mediation effect^b^	
			Estimate (SE)	P		Estimate (SE)	P		PM	P_perm_
ICAM	Carbon black exposure					−0.392 (0.168) (c)^c^	0.021			
	TNF-α (pg/ml)	94.3	−0.229 (0.069) (b)	0.0011		0.025 (0.206) (c’)	0.903		1.00	0.006
	IL-1β (pg/ml)	6.2	−0.154 (0.023) (b)	< 0.0001		−0.184 (0.154) (c’)	0.235		0.53	0.004
	IL-6 (pg/ml)	52.1	−0.076 (0.034) (b)	0.029		−0.143 (0.201) (c’)	0.478		0.64	0.05
	CRP (mg/L)	0.75	−0.028 (0.024) (b)	0.243		−0.338 (0.174) (c’)	0.054		NM	
	MIP-1β (ng/ml)	1.05	−0.065 (0.038) (b)	0.083		−0.231 (0.191) (c’)	0.230		NM	
VCAM	Carbon black exposure					−0.689 (0.227) (c)^c^	0.0027			
	TNF-α (pg/ml)	94.3	−0.313 (0.093) (b)	0.0009		−0.120 (0.278) (c’)	0.667		0.83	0.004
	IL-1β (pg/ml)	6.2	−0.016 (0.035) (b)	0.639		−0.667 (0.232) (c’)	0.0045		NM	
	IL-6 (pg/ml)	52.1	−0.123 (0.046) (b)	0.008		−0.282 (0.270) (c’)	0.297		0.59	0.038
	CRP (mg/L)	0.75	−0.063 (0.032) (b)	0.050		−0.566 (0.234) (c’)	0.016		0.18	0.092
	MIP-1β (ng/ml)	1.05	−0.083 (0.051) (b)	0.104		−0.484 (0.258) (c’)	0.062		NM	
CCL2	Carbon black exposure					−0.567 (0.221) (c)^c^	0.011			
	TNF-α (pg/ml)	94.3	−0.319 (0.090) (b)	0.0005		0.014 (0.270) (c’)	0.959		1.00	0.004
	IL-1β (pg/ml)	6.2	0.000072 (0.034) (b)	0.998		−0.567 (0.226) (c’)	0.013		NM	
	IL-6 (pg/ml)	52.1	−0.082 (0.045) (b)	0.073		−0.298 (0.265) (c’)	0.264		0.47	0.068
	CRP (mg/L)	0.75	−0.031 (0.031) (b)	0.319		−0.505 (0.229) (c’)	0.0287		NM	
	MIP-1β (ng/ml)	1.05	−0.061 (0.050) (b)	0.223		−0.417 (0.252) (c’)	0.100		NM	
CXCL8	Carbon black exposure					−3.181 (0.421) (c)^c^	< 0.0001			
	TNF-α (pg/ml)	94.3	−0.751 (0.168) (b)	< 0.0001		−1.814 (0.504) (c’)	0.0004		0.43	< 0.002
	IL-1β (pg/ml)	6.2	−0.039 (0.064) (b)	0.550		−3.129 (0.430) (c’)	< 0.0001		NM	
	IL-6 (pg/ml)	52.1	−0.347 (0.083) (b)	< 0.0001		−2.038 (0.487) (c’)	< 0.0001		0.36	< 0.002
	CRP (mg/L)	0.75	−0.070 (0.059) (b)	0.240		−3.044 (0.436) (c’)	< 0.0001		NM	
	MIP-1β (ng/ml)	1.05	−0.311 (0.092) (b)	0.0009		−2.418 (0.468) (c’)	< 0.0001		0.24	0.006
CCL5	Carbon black exposure					−0.479 (0.145) (c)^c^	0.0012			
	TNF-α (pg/ml)	94.3	−0.268 (0.058) (b)	< 0.0001		0.008 (0.174) (c’)	0.963		1.00	< 0.002
	IL-1β (pg/ml)	6.2	−0.061 (0.022) (b)	0.0057		−0.397 (0.146) (c’)	0.0071		0.17	0.01
	IL-6 (pg/ml)	52.1	−0.069 (0.030) (b)	0.020		−0.251 (0.174) (c’)	0.150		0.48	0.01
	CRP (mg/L)	0.75	−0.039 (0.020) (b)	0.054		−0.402 (0.150) (c’)	0.008		0.16	0.056
	MIP-1β (ng/ml)	1.05	−0.104 (0.032) (b)	0.0014		−0.224 (0.162) (c’)	0.168		0.53	0.002

*CBP* Carbon black packer, *IQR* Inter-quartile range, *SE* Standard error, *GLM* Generalized linear model

^a^GLM was used to assess the association (c’) between carbon black exposure and delta Ct of selected biosensor genes with age, overweight and obesity, current smoking status, packyears, passage of cells, and cytokines or chemokines (b) included as covariate for adjustment. Non-transformed data of cytokine and chemokine levels were used in GLM. c’ and b should be referred to Fig. 1

^b^The proportion mediated effect size that quantifies the proportion of a total effect mediated was calculated using the following equation: (c-c’) / c. The database was permuted for 500 times to generate a null distribution of c-c’. P_perm_ was calculated as the number of permuted databases generating a c-c’ that is less than observed value divided by 500. c should be referred to Fig. 1

^c^GLM was used to assess the association between carbon black and delta Ct of selected biosensor genes in 106 non-CBPs and 82 CBPs with adjustment for age, overweight and obesity, current smoking status, and packyears, and passage of cells. c should be referred to Fig. 1

### In vitro intervention study

**Fig. 2:**
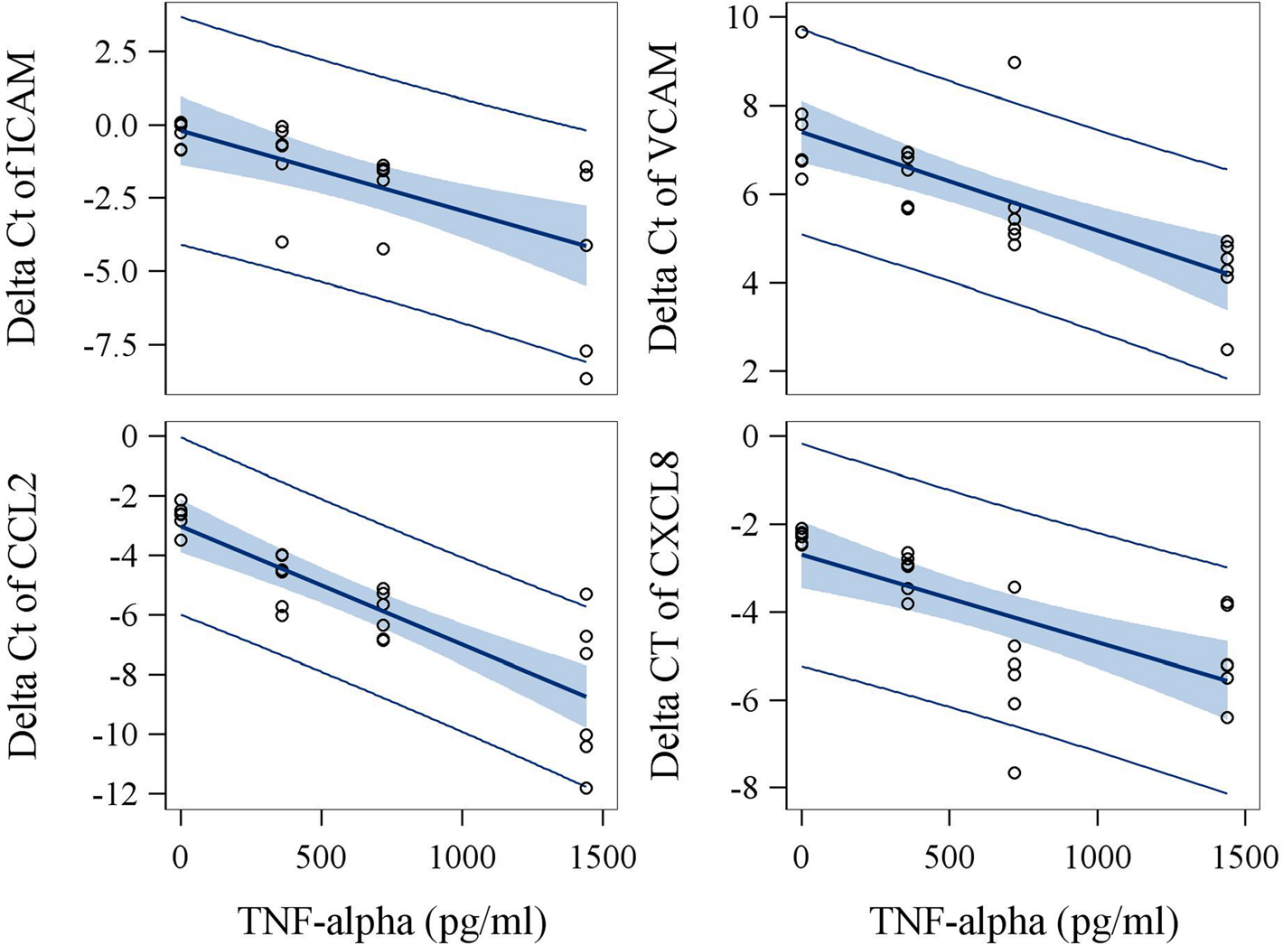
The effect of TNF-α on expression (delta Ct) of biosensor genes in vitro using hUVECs. Serum samples obtained from three workers with lowest levels of TNF-α were spiked with exogenous TNF-α at 0, 360, 720 and 1440 pg/ml as final concentrations. The highest concentration of added TNF-α is about 2-fold higher than the highest level seen in study subjects. Cultures of biosensor assay were conducted in duplicates. The slopes were listed as estimates in supplemental Table 3. Among the seven biosensor genes studied, ICAM, VCAM, CCL2, and CXCL8 expressions were identified to be dramatically induced by TNF-α treatment. Relative quantification ranged from 1.88 for CXCL8 to 3.05 for CCL2 per 500 pg/ml addition of TNF-α in the culture medium

**Fig. 3:**
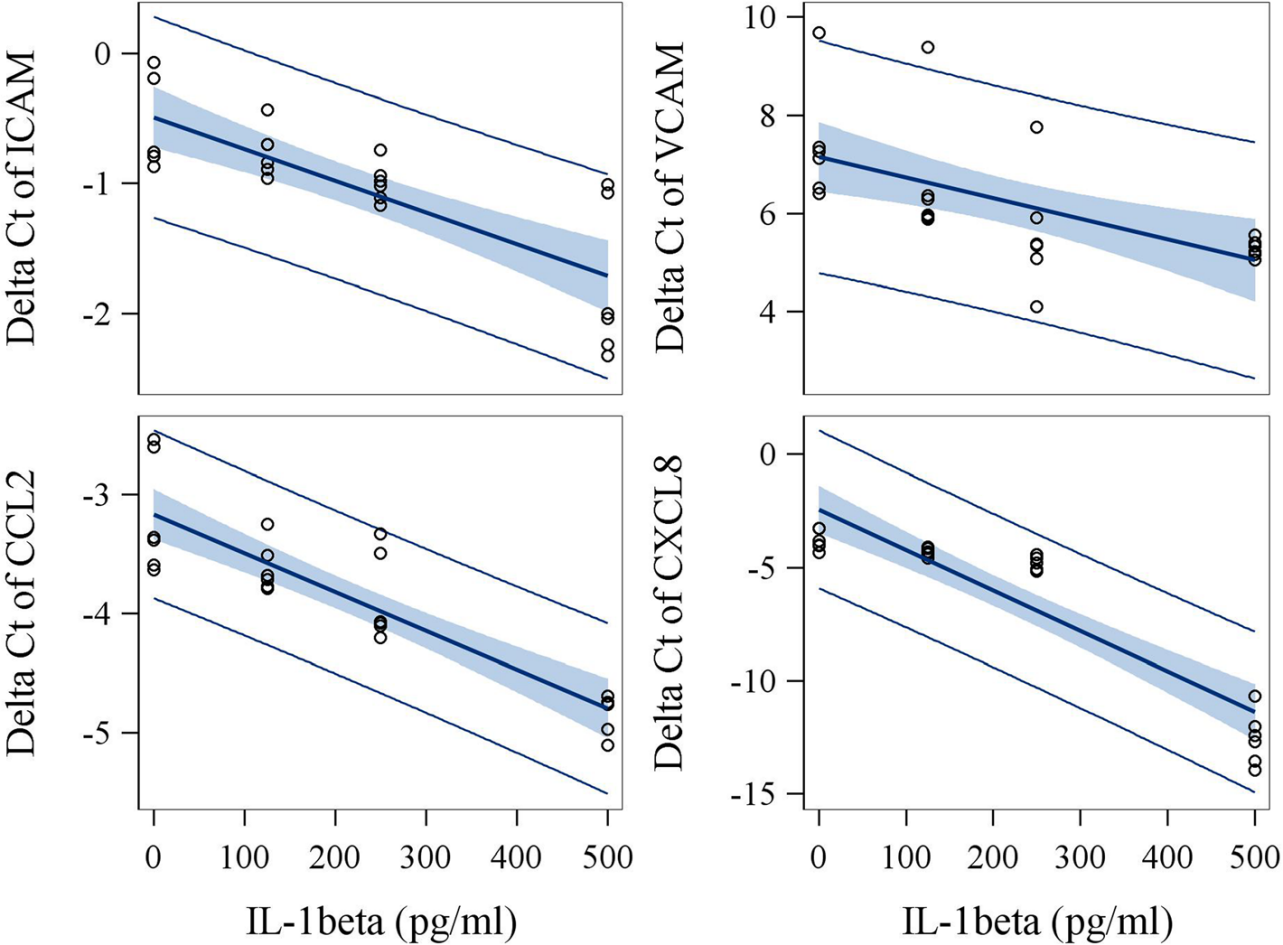
The effect of IL-1β on expression (delta Ct) of biosensor genes in vitro using hUVECs. Serum samples obtained from three workers with lowest levels of IL-1β were spiked with exogenous IL-1β at 0, 125, 250 and 500 pg/ml as final concentrations. The highest concentration of added IL-1β is about 2-fold higher than the highest level seen in study subjects. Cultures of biosensor assay were conducted in duplicates. The slopes were listed as estimates in supplemental Table 3. Among the seven biosensor genes studied, ICAM, VCAM, CCL2, and CXCL8 expressions were identified to be dramatically induced by IL-1β treatment. Relative quantification ranged from 2.43 for CCL2 to 349.71 for CXCL8 per 500 pg/ml addition of IL-1β in the culture medium

We further conducted an in vitro intervention experiment in which various concentrations of TNF-α, IL-1β, IL-6, MIP-1β and CRP were applied to endothelial cells to assess the relationship between concentrations of these mediators and the magnitude of increased expression of biosensor genes. Physiologically relevant concentrations for these 5 mediators were selected based on circulating levels seen in studied subjects. Addition of IL-1β and TNF-α to subjects’ media with the lowest background level for tested factors significantly increased mRNA expression of ICAM, VCAM, CCL2, and CXCL8 in primary hUVECs (Figs. 2 and 3, Supplemental Table 3). We also found that the addition of IL-6 to the medium significantly increased mRNA expression of CCL5 in hUVEC (Supplemental Figure 1, Supplemental Table 3). The potency of inducing CXCL8 gene expression was significantly higher for IL-1β than TNF-α in vitro (*P* < 0.0001). In addition, although responses varied among the three subjects probably due to background levels of other cofactors in serum, the slopes of linear curves between levels of added cytokines or chemokines and gene expression (delta Ct) were similar among three serum samples from different individuals. Importantly, the induction of biosensor gene expression by TNF-α (for ICAM, VCAM, CCL2, and CXCL8), IL-1β (for ICAM), and IL-6 (for CCL5) observed in in vitro studies was highly consistent with the results of the mediation analyses at the population level (Table 4). Specifically, the magnitude of associations of biosensor ICAM and CCL2 with TNF-α, and of biosensor CCL5 with IL-6 in the in vitro studies were almost identical to that seen in the population study (Supplemental Table 3). Inspection of dose-response curves (Figs. 2 and 3) identified that the induction of CCL2 expression by the addition of IL-1β or TNF-α showed the least variation across sera from different subjects and higher proportion of variance explained by the model (R^2^ = 0.95 for IL-1β and R^2^ = 0.76 for TNF-α). Thus, based on the dose-response curves, the difference of CCL2 expression between CBPs and non-CBPs (estimate = − 0.57, Table 2) was equivalent to the effect caused by an increase of 177.5 pg/ml TNF-α or 222.9 pg/ml IL-1β in circulation.

## Discussion

In this study, the methodology of an established biosensor assay was further advanced by the creation of a global score quantifying the magnitude of endothelial cell activation that was extracted from mRNA expression of seven biosensor genes using principal component analysis and was highly responsive to carbon black exposure. Our study provides strong evidence of ex vivo activation of coronary artery endothelial cells caused by inflammation mediators in serum from CBPs as a result of chronic inhalation exposure to high levels of carbon black nanoparticles. Moreover, an in vitro intervention study estimated that the expression difference of the biosensor gene CCL2 between CBPs and non-CBPs was equivalent to the effect caused by an increase of 177.5 pg/ml for TNF-α or 222.9 pg/ml for IL-1β in circulation.

Inflammatory activation of endothelial cells following stress or injury is essential to both early vascular disease initiation as well as progression of advanced plaques [19]. By assessing the expression of adhesion molecules and chemotactic factors in hCAECs in vitro, this biosensor assay provided a holistic approach to quantify the capacity of serum samples from subjects with a specific exposure to induce endothelial cell activation. This assay has been shown to outperform established serum inflammation markers in discriminating patients with coronary artery diseases from healthy controls [21]. Although endothelial cell activation may be reversible, chronic and persistent endothelial cell activation may progress to irreversible stages including endothelial apoptosis and necrosis that result in impaired endothelial barrier integrity and endothelial dysfunction which have been recognized as major pathological changes underlying vessel diseases seen in patients with hypertension or diabetes [19, 26]. An interaction of borderline significance (*P* = 0.072) between carbon black exposure and overweight or obesity for affecting CXCL8 expression in hCAECs was observed in this study. Stratification analysis showed a significantly increased level of CXCL8 expression associated with overweight or obesity in CBPs (RQ = 2.58, *P* = 0.046), but not in controls (*P* = 0.57). Thus, it is likely that chronic exposure to a high level of carbon black aerosol in CBPs could induce persistent endothelial cell activation in situ that may make CBPs more susceptible for acquisition of cardiovascular diseases when they also acquire established risk factors such as metabolic syndrome, an hypothesis that warrants future testing.

The ex vivo nature of the biosensor assay allows for interrogation of the role of inflammatory mediators of interest by directly modulating their levels in serum samples. In this study, candidate mediators were identified by conducting mediation analyses that statistically addressed the magnitude of association between carbon black exposure and biosensor response that could be explained by the inclusion of mediators in the model. The combination of mediation analyses and in vitro functional validation confirmed TNF-α, IL-1β, and IL-6 as important circulatory factors mediating the effects of carbon black exposure on biosensor responses. Most importantly TNF-α, with largest increase in CBPs versus controls, had complete mediation effect for CCL2, CCL5, ICAM, and VCAM expressions, and partial mediation effect for CXCL8 expression. In vitro intervention also confirmed a direct effect of TNF-α on CCL2, ICAM, VCAM, and CXCL8 expression in hUVECs. These findings strongly support the premise that circulatory inflammation, probably with TNF-α as the major player, mediated the extra-pulmonary effects caused by carbon black exposure in occupational workers. Additional support for this mode of effect came from our study that carbon black exposure could increase the genomic instability in peripheral lymphocyte in a dose-dependent manner and this association could be again completely mediated by TNF-α in circulation [16].

The major cellular origin of serum TNF-α in carbon black exposed workers is unclear. Our previous study analyzed the correlation between blood leucocytes, lymphocytes and their subsets and circulatory cytokines and chemokines and did not identify any association with serum TNF-α levels, suggesting blood leucocytes are not major sources for generating TNF-α in circulation [24]. Study showed that in vitro treatment of alveolar macrophages with ambient urban particles and carbon black could induce particle phagocytosis and stimulate a dose-dependent secretion of TNF-α in supernatant [27]. Moreover, studies using C57BL/6 mice intratracheally exposed to a single instillation of inhalable particulate matters has shown that IL-6 as a surrogate for inflammation markers produced in the lung can directly migrate into the systemic circulation and contribute to the vascular dysfunction of the abdominal aorta [28]. In addition, the majority of airway macrophages stained positive for IL-6 contained carbon particles. A 90-day nose only exposure study of Sprague–Dawley rats exposed to 30 mg/m^3^ carbon black aerosol for 6 h per day identified a dramatic increase of TNF-α in lung supernatant which sustained for 14 days after the 90-day exposure [29]. Thus, we hypothesize that alveolar macrophages may be the main source for TNF-α in the lungs after carbon black exposure which could be translocated to systemic circulation in a pattern similar to the one observed for IL-6 in the mouse model. Importantly, other factors not characterized in the present analysis may also drive pathologic responses in endothelial cells. Recent findings with pulmonary exposure to multi-walled carbon nanotubes revealed thousands of bioactive, fragmented endogenous peptides in the serum that appeared to arise from metalloproteinase activity in the lung [30, 31]. Thus, the role of traditional mediators like TNF-α may be augmented by other factors shed from the exposed lung to further promote chronic vascular disease.

## Conclusions

In summary, our studies provide a proof of concept that as one category of poorly soluble, widely used nanoparticles, exposure to carbon black via inhalation may execute its extra-pulmonary effects (e.g., endothelial cell activation and genomic instability) through elevating systemic inflammation with TNF-α as a potential pivotal mediator. In addition, our findings also support the premise that inhalation of carbon black aerosol may be a risk factor for subsequent development of vessel diseases especially when comorbidity exists. Our research could serve as a benchmark study to disentangle the role of different constituents in ambient particulate matter and of engineered carbon-based nanoparticles in causing extra-pulmonary toxicity and health effects.

## Methods

### Study subjects

The protocol was approved by the Research Ethics Committee of the National Institute for Occupational Health and Poison Control, Chinese Center for Disease Control and Prevention. Design details including inclusion and exclusion criteria were published before [14, 15]. Briefly, the CBP study was established in 2012 by recruiting male CBPs who have bagged newly manufactured carbon black for more than 6 months and male non-CBP controls from a local water authority with no specific exposure to carbon black (Table 1). Written informed consent was acquired from all participants prior to the interview and any procedures.

### Exposure assessment

Physicochemical characteristics of carbon black particles, ambient levels of carbon black aerosol (e.g., PM2.5, PM2.5 related elemental carbon, organic carbon, and total carbon) inside the bagging facilities and reference areas, and particle size distributions of carbon black aerosol inside the bagging facilities have been reported in our previous studies [14–16, 24]. Urinary hydroxyl metabolites of six polycyclic aromatic hydrocarbons were determined using HPLC-MS/MS [32].

### Cell culture and biosensor assay

Primary hCAECs (ATCC PCS-100-020) between passages 4 and 6 were grown to confluence in 24-well plates in vascular cell basal media (ATCC PCS-100-030) supplemented with endothelial cell growth kit-BBE (ATCC PCS-100-040). Cells were serum-starved with basal media for 24 h prior to the incubation in basal medium spiked with 10% serum obtained from study subjects for 4 h at 37 °C. Each plate of cells was treated with a proportional number of non-CBPs and CBPs in a randomized and blind fashion. mRNA expression levels of seven target genes including CCL2 (Hs00234140_m1), CCL5 (Hs00982282_m1), CXCL8 (Hs00174103_m1), CXCL12 (Hs00171022_m1), ICAM (Hs00164932_m1), SELP (Hs00174583_m1), and VCAM (Hs01003372_m1), and one endogenous control gene (TBP, Hs00427620_m1) were measured using TaqMan assay (ThermoScientific Applied Biosystems). These seven genes were selected based on the key role of expressed proteins on the surface of endothelial cells upon activation for chemotaxis and adhesion of leukocytes [19]. Two serum pools were created by combining serum aliquots from 15 non-CBPs and 10 CBPs, respectively and were assayed with each batch of experiments as quality assessment samples for 20 and 12 times, respectively. Coefficients of variation for Cts of eight genes ranged from 2.8 to 11.1% with an average of 6.7%. In addition, Cts of TBP were not associated with smoking status, packyears, and carbon black exposure status, further supporting its appropriateness as an endogenous control gene. The average RNA yield from hCAEC cultures treated with sera from non-CBPs and CBPs was very similar (Table 1).

### CXCL8 protein level in medium

Supernatants were collected for the first 52 samples (19 CBPs and 33 non-CBPs) which provided sufficient power for assessing the correlation between mRNA expression and secreted protein of CXCL8. CXCL8 levels in the supernatants were analyzed by CXCL8 human uncoated enzyme-linked immunosorbent assay Kits (ThermoScientific Invitrogen). Net increase of CXCL8 level in supernatants was calculated by subtracting 10% serum level from supernatant level.

### Circulatory inflammatory markers

Three pro-inflammatory cytokines (i.e., IL-1β, IL-6, and TNF-α) and two chemokines (i.e., CXCL8 and MIP-1β) were measured in serum using cytometric bead array (BD Biosciences, USA) [14]. Serum C-reactive protein (CRP) was measured with an immunoturbidimetric assay (DiaSys, Germany) [14].

### In vitro intervention study

Biologically verified primary human umbilical vein endothelial cells (hUVEC) were used to assess the effect of selected cytokines and chemokines on gene expression. A similar procedure for the biosensor assay as described above was followed except that hUVECs were serum-starved for 2 h due to elevated sensitivity. Serum samples obtained from three workers with lowest levels of targeted cytokines were spiked with different concentrations of cytokines and chemokines for interference. The interference cytokines and chemokines were determined by mediation analysis, and included CRP (0, 12.5, 25 and 50 μg/mL; Sino Biological, 11,250-HNAH), TNF-α (0, 360, 720 and 1440 pg/ml; Pepro Tech., 300-01A), IL-1β (0, 125, 250 and 500 pg/ml; Pepro Tech., 200-01B), IL-6 (0, 430, 860 and 1720 pg/ml; Pepro Tech., 200–06), and MIP-1β (0, 1.25, 2.5 and 5 ng/ml, Sino Biological, 10,899-H08Y). The highest concentration of added cytokines is about 2-fold higher than the highest level seen in the serum of workers.

### Statistical analysis

First, generalized linear model (GLM) was used to assess the association between carbon black exposure and hCAECs activation with adjustment for age, overweight and obesity, current smoking status, packyears, and passage of cells. Principal component analysis was conducted based on delta Cts of seven biosensor genes to extract a major PC defining a global score quantifying the magnitude of hCAECs activation which was assessed in association with carbon black exposure to minimize the effect of multiple comparisons. Individual gene association with carbon black exposure was further assessed with delta Ct as the outcome. Principal component analysis identified two PCs with each explaining a proportion of total variance larger than average (1/7 = 0.142, Supplemental Table 1). Thus, we applied two independent analyses in calculating Bonferroni corrected *P* values (0.05/2 = 0.025) for claiming a significant association in individual gene analyses. Second, the difference of magnitude of the association between models with (c’) and without (c) adjustment for circulatory inflammation (Fig. 1a) was calculated to quantify the mediation effect of circulatory inflammatory factors on the association between carbon black exposure and hCAECs activation. A permutation-based method was used to assess whether the proportion mediated was statistically significant or not and was entailed in figure legend of Fig. 1b. Third, GLM was used to quantify the change of expression (delta Ct) of biosensor genes by TNF-α, IL-1β, or IL-6 treatments in media in an in vitro intervention study with adjustment for serum ID. An interaction term between treatments and serum ID was included in the GLM to assess whether the slopes of dose-response were different among the three individuals. Plots were created using SAS ODS Graphics Designer and R × 64 3.6.1. All statistical analyses were conducted using SAS (version 9.4, NC, USA, site 70,239,492 and site 70,080,753).

## Supplementary information

**Additional file 1.**

## Data Availability

Detailed assay protocols, SAS codes, and datasets used and/or analyzed during the current study are available from the corresponding author on reasonable request.
