# Supplementary material for: Occupational exposure to carbon black nanoparticles increases inflammatory vascular disease risk: an implication of an ex vivo biosensor assay
Source: Part Fibre Toxicol. 2020 Sep 29;17:47. doi: 10.1186/s12989-020-00378-8 (PMC7523398; doi:10.1186/s12989-020-00378-8)
Supplement: Supplementary file 1 — Additional file 1. [file 12989_2020_378_MOESM1_ESM.docx]

Supplemental materials

**Occupational exposure to carbon black nanoparticles increases inflammatory vascular disease risk: an implication of an *ex vivo* biosensor assay**

Jinglong Tang^1^, Wenting Cheng^1^, Jinling Gao^1^, Yanting Li^1^, Ruyong Yao^2^, Nathaniel Rothman^3^, Qing Lan^3^, Matthew J. Campen^4^, Yuxin Zheng^1#^, Shuguang Leng^1,5,6#^

Supplemental Table 1. Biosensor principal component analysis in 106 non-CBPs and 82 CBPs (delta Ct)

| Variable | PC1 | PC2 | PC3 | PC4 | PC5 | PC6 | PC7 |
| --- | --- | --- | --- | --- | --- | --- | --- |
| CCL2 | **0.45** | -0.13 | -0.02 | -0.37 | 0.48 | 0.59 | -0.25 |
| CCL5 | **0.43** | 0.09 | 0.06 | -0.59 | -0.65 | -0.18 | -0.07 |
| CXCL8 | **0.36** | -0.28 | 0.54 | 0.52 | -0.34 | 0.33 | 0.12 |
| CXCL12 | 0.20 | **0.66** | -0.44 | 0.25 | -0.18 | 0.40 | 0.27 |
| ICAM | **0.45** | -0.003 | -0.30 | 0.43 | 0.06 | -0.39 | -0.60 |
| SELP | 0.10 | **0.66** | 0.64 | -0.02 | 0.30 | -0.21 | -0.11 |
| VCAM | **0.48** | -0.15 | -0.09 | -0.02 | 0.34 | -0.39 | 0.69 |
| Total variance explained | 0.41 | 0.18 | 0.12 | 0.09 | 0.07 | 0.06 | 0.05 |

CBP: carbon black packer; PC = principal component

Principal component analysis was conducted to extract the major principal components. The first two principal components explained proportion of total variance larger than average (1/7=0.142). PC1 has loading from CCL2, CCL5, CXCL8, ICAM, and VCAM. PC2 has loading from CXCL12 and SELP.

Supplemental Table 2. Differential effects of carbon black exposure on pro-inflammatory cytokines and chemokines in blood ^a^

| Variable ^b^ | Non-CBPs ^c^ | CBPs ^c^ | Ratio (95%CI) ^d^ | P |
| --- | --- | --- | --- | --- |
| IL-6 (pg/ml) | 23.1 (11.7 - 63.8) | 158.0 (86.9 - 288.7) | 7.0 (4.6 - 10.8) | <0.0001 |
| TNF-α (pg/ml) | 14.8 (12.5 - 106.8) | 141.0 (133.7 - 299.4) | 10.2 (4.0 - 25.9) | <0.0001 |
| IL-8 (ng/ml) | 0.71 (0.50 - 1.1) | 1.0 (0.91 - 2.2) | 1.3 (0.88 - 2.0) | 0.17 |
| MIP-1β (ng/ml) | 0.81 (0.45 - 1.5) | 2.6 (1.9 - 4.3) | 3.3 (2.6 - 4.2) | <0.0001 |
| IL-1β (pg/ml) | 0.64 (2.0 - 8.2) | 6.6 (5.9 - 20.4) | 10.6 (3.5 - 32.4) | <0.0001 |
| MCP1 (ng/ml) | 0.24 (0.19 - 0.32) | 0.22 (0.18 - 0.29) | 0.94 (0.82 - 1.1) | 0.37 |
| CRP (mg/L) | 0.48 (0.25 - 1.0) | 1.20 (0.66 - 1.6) | 2.7 (1.9 - 3.8) | <0.0001 |

CBP: carbon black packer; CI = confidence interval; GM = geometric mean; Q = quartile; BMI = body mass index; GLM = generalized linear model

^a^ GLM was conducted to examine the association between carbon black exposure and the outcome variables with adjustment for age, BMI, current smoking and alcohol use status, and packyears. Independent variables were natural log transformed. This analysis included 106 non-CBP controls and 85 CBPs.

^b^ Unit was pg/ml for IL-6, TNF-α, and IL-1β, ng/ml for IL-8, MIP-1β, and MCP1, and mg/L for CRP.

^c^ GM (Q1 - Q3).

^d^ Ratio (95% CIs) was calculated from the regression coefficient for an explanatory variable in the GLM, β, through the equation ratio=e^β^.

Supplemental Table 3. The effect of TNF-α, IL-1β, and IL-6 on expression of individual biosensor genes from the *in vitro* intervention study

| Biosensor gene | Variable | In vitro study | | | | |  | Extrapolation to the population study | |
| --- | --- | --- | --- | --- | --- | --- | --- | --- | --- |
|  |  | Unit of change | Estimate (SE)^a^ | RQ^b^ | P^a^ | P_int_^c^ |  | IQR in non-CBPs | Estimate (SE) |
| ICAM | TNF-α (pg/ml) | 500 | -1.04 (0.42) | 2.06 | 0.023 | 0.84 |  | 94.3 | -0.196 (0.079) |
| ICAM | IL-1β (pg/ml) | 500 | -1.52 (0.24) | 2.87 | <0.0001 |  |  | 6.2 | -0.019 (0.003) |
| VCAM | TNF-α (pg/ml) | 500 | -0.94 (0.37) | 1.92 | 0.021 | 0.12 |  | 94.3 | -0.177 (0.070) |
| VCAM | IL-1β (pg/ml) | 500 | -2.59 (1.06) | 6.02 | 0.025 |  |  | 6.2 | -0.032 (0.013) |
| CCL2 | TNF-α (pg/ml) | 500 | -1.61 (0.47) | 3.05 | 0.0031 | 0.55 |  | 94.3 | -0.304 (0.089) |
| CCL2 | IL-1β (pg/ml) | 500 | -1.28 (0.17) | 2.43 | <0.0001 |  |  | 6.2 | -0.016 (0.002) |
| CXCL8 | TNF-α (pg/ml) | 500 | -0.91 (0.42) | 1.88 | 0.044 | <.0001 |  | 94.3 | -0.172 (0.079) |
| CXCL8 | IL-1β (pg/ml) | 500 | -8.45 (1.61) | 349.71 | <0.0001 |  |  | 6.2 | -0.105 (0.020) |
| CCL5 | IL-6 (pg/ml) | 500 | -0.67 (0.24) | 1.59 | 0.0125 |  |  | 52.1 | -0.070 (0.025) |

CBP = carbon black packer; RQ = relative quantification; IQR = inter-quartile range; SE = standard error; GLM = generalized linear model

^a^ GLM was used to quantify the associations between biosensor gene expressions (delta Ct) and addition of TNF-α, IL-1β, or IL-6 in the medium in *in vitro* intervention study with adjustment for serum ID. The slopes of linear curves between gene expression (delta Ct) and levels of added cytokines or chemokines were of no difference between serum samples from three individuals.

^b^ Relative quantification was calculated as RQ=2^-(estimate)^.

^c^ P_int_ represents the significance for the differences of slopes of inducing expression (Delta Ct) of biosensor genes between TNF-α and IL-1β treatments with adjustment for serum ID.

Supplemental figure 1.


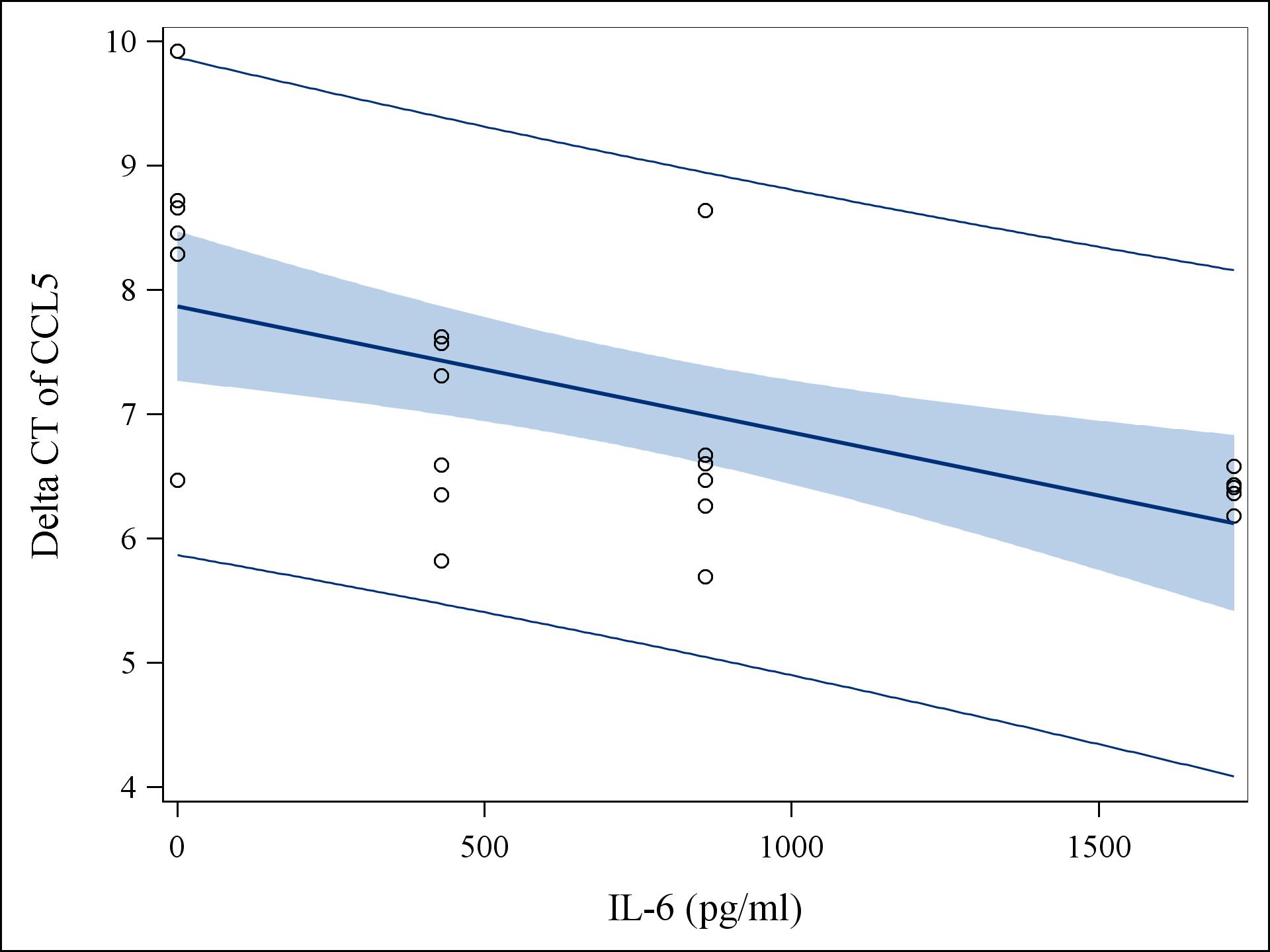


**The effect of IL-6 on expression (delta Ct) of biosensor genes CCL5** *in vitro* using endothelial cells isolated from human umbilical cord blood vessel. The slopes were listed in supplemental table 3. Every 500 pg/ml increase of IL-6 in medium caused a 59% increase in relative quantification of CCL5 expressed in hUVEC.
